# Preliminary evidence of safety and effectiveness of Loxoprofen Sodium Cataplasm combined with physiotherapy for myofascial pain syndrome treatment: A randomized controlled pilot clinical trial

**DOI:** 10.3389/fneur.2022.998327

**Published:** 2022-11-22

**Authors:** Xuewen Zhou, Xuelian Li, Ziyang Wang, Dong Huang

**Affiliations:** ^1^Department of Pain, The Third Xiangya Hospital and Institute of Pain Medicine, Central South University, Changsha, China; ^2^Department of Anesthesiology, Sun Yat-sen Memorial Hospital, Sun Yat-sen University, Guangzhou, China; ^3^Hunan Key Laboratory of Brain Homeostasis, Central South University, Changsha, China

**Keywords:** myofascial pain syndrome, Loxoprofen Sodium Cataplasm, physiotherapy, extracorporeal shock wave therapy, transcutaneous electrical nerve stimulation

## Abstract

**Background:**

Myofascial pain syndrome (MPS) is one of the most common causes of chronic skeletal muscle pain, which is closely related to skeletal muscle myofascial trigger point (MTRP). Since there is no first-line treatment for MPS, we investigated Loxoprofen Sodium Cataplasm combined with physiotherapy as a non-invasive therapy in patients at different levels to a protocol with superior efficacy that is safe and easy to promote. Moreover, this treatment could represent an alternative therapeutic strategy for low-income patients to a safer, more convenient, and more economical treatment scheme.

**Methods:**

A randomized clinical study was aimed at evaluating the safety and efficacy of Loxoprofen Sodium Cataplasm combined with physiotherapy in patients diagnosed with MPS in the pain clinic. We screened 100 patients with MPS, and using a computer-generated random allocation sequence, we stratified patients in a ratio of 2:1:1:1 (A: B: C: D) to one of the four treatment groups. Group A received Loxoprofen Sodium Cataplasm combined with extracorporeal shock wave therapy (ESWT) and transcutaneous electrical nerve stimulation (TENS). Group B received Loxoprofen Sodium Cataplasm alone. Group C received physiotherapy alone. Group D received Flurbiprofen Cataplasm combined with physiotherapy. After 2 weeks of treatment, the overall efficiency and secondary assessment indicators, including visual analog scale (VAS) scores, chronic soft tissue injury (CSTI) scores, Oswestry Disability Index (ODI) scores, or Northwick Park Neck Pain Questionnaire (NPQ) scores, were evaluated before and after treatment to analyze the difference in efficacy of each group.

**Results:**

All groups were well tolerated with no reported adverse events. Significant treatment differences in the change from baseline in overall efficiency (primary efficacy endpoint) (*P* = 0.0078) were observed in subjects of groups A and C.

**Conclusion:**

Showing valuable data of efficacy in primary and secondary endpoints, Loxoprofen Sodium Cataplasm combined with physiotherapy is superior in the treatment of MPS.

**Trial registration number:**

https://www.chictr.org.cn/ (ChiCTR2100054756).

## Introduction

Myofascial pain syndrome (MPS) is one of the most common causes of chronic musculoskeletal pain. The main symptoms are asymmetric pain at the trigger point, local or systemic, and even some patients have symptoms of autonomic dysfunction, including flushing, lacrimation, dermatographia, diaphoresis, goose rash, and dizziness (1, 2). There are numerous treatment options available for MPS, mainly including oral non-steroidal anti-inflammatory drugs (NSAIDs), opioids, benzodiazepine sedatives, muscle relaxants, tricyclic antidepressants, topical drugs, moderate aerobic exercise, trigger point injections, transcutaneous electrical nerve stimulation (TENS), extracorporeal shock wave (ESWT), acupuncture, dry needling, botulinum toxin injection, Kinesio taping, and hot compress, but no treatment with significant clinical advantages has been summarized (2–6). In the treatment of MPS, first-line NSAIDs are widely used clinically and play an important role in the treatment of MPS (7). NSAIDs have definite therapeutic effects on pain mainly by inhibiting cyclooxygenase, reducing the synthesis of prostaglandins, reducing the stimulation of afferent nerve endings, and preventing the release of pain-causing substances (8). However, long-term use of NSAIDs and oral preparations may cause adverse reactions in the gastrointestinal tract and even the whole body. In severe cases, life-threatening side effects such as gastrointestinal bleeding and abnormal cardiac function may occur. In addition, some invasive treatment methods, such as dry needling and local injection have definite curative effects, are expensive, and may have adverse reactions such as bleeding and infection (9–16). In addition, limited by regional differences in medical level, invasive treatment is not conducive to widespread promotion.

Myofascial pain syndrome has a huge patient population, with a prevalence of up to 55–90% in pain clinics, and is prone to recurrence after treatment (2, 17, 18), causing a huge burden on society and individuals. Therefore, the unified use of a non-invasive, safe, effective, inexpensive, and popularized outpatient treatment plan is crucial.

Currently, topical analgesics such as Loxoprofen Sodium Cataplasm, Flurbiprofen Cataplasm, and Compound Methyl Salicylate Cataplasm are often used in the treatment of MPS in pain clinics. Physical therapy such as ESWT/TENS is also used. There is also a combination of the two treatment options, but there is no clear and more effective treatment.

Therefore, this study used the visual analog scale (VAS) scores, Oswestry Disability Index (ODI) scores, and NPQ scores as evaluation indicators to explore the efficacy and safety of different regimens in the treatment of MPS and to provide a safer, more effective, more convenient, and less expensive treatment plan for the treatment of MPS.

## Subjects and methods

### Research design

From 1 August 2021, to 31 December 2021, outpatients in the Pain Department of the Third Xiangya Hospital of Central South University aged 18–75 years were screened and diagnosed with MPS according to diagnostic criteria (7, 19). After excluding patients with pregnancy, fractures, liver and kidney insufficiency, immune diseases, using NSAIDs, opioids, glucocorticoids, receiving physical therapy within 1 month, and allergic to NSAIDs, patients included in the study were classified into 4 groups by random number table method. This study was reviewed and approved by the Clinical Trial Ethics Committee of the Third Xiangya Hospital of Central South University (No. 21136), and all patients signed informed consent. This trial was registered on www.chictr.org.cn/ with the following number: ChiCTR2100054756.

Group A received Loxoprofen Sodium Cataplasm combined with physiotherapy, and 40 patients were planned to be included. Physiotherapy used extracorporeal shock wave therapy (ESWT) and TENS. Group B received Loxoprofen Sodium Cataplasm alone, and 20 patients were planned to be included. Group C received physiotherapy alone, and 20 patients were planned to be included. Group D received Flurbiprofen Cataplasm combined with physiotherapy, and 20 patients were planned to be included.

In groups A, B, and D, the method of external drug use is 1 cataplasm once per day, stick to the painful area; each application exists 12 h to 24 h; and the course of treatment is 2 weeks. In groups A, C, and D, physiotherapy consisted of ESWT and TENS, once a week, with a total of two times, i.e., on the day of enrollment and 1 week after enrollment, and no topical drug was used on the day of treatment. The frequency of physiotherapy was once a week, the same as described by Kiraly et al. (20).The parameters of ESWT were 1,000 impulses, 1.5 bar, 10 Hz, energy density of 0.25 mJ/mm^2^, and 15 mm treating head diameter to the trigger point and its vicinities. The parameters of TENS were 100 Hz, pulse duration (width) of 250 μs, and treatment for 15 min. Patients in groups A, C, and D were treated with the same model, operated by the same physician, in the Pain Department of the Third Xiangya Hospital of Central South University. In addition, health education was provided to each enrolled patient, including moderate exercise, attention to the intensity of work, and correction of poor posture.

### Assessment and follow-up

Main evaluation indicators are total effective rate: the VAS-weighted value was used to determine the curative effect, i.e., VAS-weighted value = (VAS scores before treatment-VAS scores after treatment)/VAS scores before treatment × 100%; recover: VAS weighted 75–100%; evident: VAS weighted 50–75%; effective: VAS weighted 25–50%; effectless: VAS weighted <25%.

Secondary evaluation indicators are (1) VAS scores, (2) chronic soft tissue injury (CSTI) scores, and (3) ODI scores or NPQ scores. The VAS scores range from 0 to 10, and are based on self-reporting with the VAS; lower scores indicate less pain. The CSTI score covers the degree of pain, the size of palpation of the taut band and/or nodule, and the functional status; lower scores indicate less pain, smaller taut band or nodule, and better functional status. The ODI assesses the change in the functional status of adults with low back pain. The ODI contains ten pain-related questions scored from zero (no pain) to five (most severe pain). Scores are expressed as a percentage of total points; lower scores indicate better functional status (21). The Northwick Park Neck Pain Questionnaire (NPQ), which measures the level of neck pain and the resulting disability, is a nine-item questionnaire with five possible responses for each question; lower scores indicate less neck pain and better functional status (22). All the above indicators were evaluated before treatment and on the 15th day after treatment.

### Statistical methods

Differences in overall efficiency were compared using χ^2^ tests (e.g., possible χ^2^-corrected tests and Fisher's exact test), and non-parametric tests (Mann–Whitney U test) were used to compare differences in clinical symptom improvement rates and patient compliance between groups. Analyses were performed using IBM SPSS Statistics 24 (version 24.0.0.0) at a significance level of *P* < 0.05.

## Results

### Patient grouping flowchart and general semographics

We included a total of 94 patients, including 34 in group A, 21 in group B, 19 in group C, and 20 in group D; the flowchart of patient grouping is shown in Supplementary Figure 1. The general demographics are shown in Table 1. The results showed that there was no significant difference in the age, course of the disease, and gender distribution of the patients in each group.

**Table 1:** General characteristics of patients.

	**Group A** **(*N =* 34)**	**Group B** **(*N =* 21)**	**Group C** **(*N =* 19)**	**Group D** **(*N =* 20)**	***P-value***
Age (years)
*n*	34	21	19	20	
Mean (SD)	40 (15)	38 (11)	40 (15)	40 (15)	0.8858^a^
Median (P_25_,P_75_)	40 (25, 54)	35 (30, 47)	37 (29, 51)	40 (31, 47)	
Min, Max	18, 68	22, 59	18, 76	21, 77	
Age group-*n* (%)
Young (age <35)	12 (35.3)	10 (47.6)	9 (47.4)	9 (45.00)	0.7335^b^
Middle-aged (35 ≤ age <60)	18 (52.9)	11 (52.4)	9 (47.4)	9 (45.00)	
Elderly (age ≥ 60)	4 (11.8)	0 (0.0)	1 (5.3)	2 (10.00)	
Gender-*n* (%)
Male	15 (45.45)	5 (23.81)	11 (57.89)	12 (63.16)	0.0606^b^
Female	18 (54.55)	16 (76.19)	8 (42.11)	7 (36.84)	
Disease course group-*n* (%)
<2 years	29 (85.29)	16 (76.19)	13 (68.42)	15 (75.00)	0.2500^b^
≥2 years	5 (14.71)	5 (23.81)	6 (31.58)	5 (25.00)	

Groups A and D are each missing one gender data. a: *T*-test was used for *p-*values. b: Chi-square test was used for *p*-values.

### Intergroup comparison of overall efficiency in each group

As shown in Table 2, the results show that each treatment method has a certain effect on patients with MPS. There was no significant difference in overall efficiency between group A vs. group B and group A vs. group D (*P* > 0.999, *P* = 0.7657), which was not statistically significant. There were significant differences between group A vs. group C, and group B vs. group C (*P* = 0.0078, *P* = 0.0262), with statistical significance. It is suggested that the combination of Loxoprofen Sodium Cataplasm and physiotherapy or Loxoprofen Sodium Cataplasm alone is better than physiotherapy alone.

**Table 2:** Effective number of people in each group (overall efficiency) *n* (%).

**Test groups**	**Recover**	**Evident**	**Effective**	**Effectless**	**Overall efficiency**
Group A (*N =* 34)	8 (23.53)	9 (26.47)	11 (32.35)	6 (17.65)	28 (82.35)
Group B (*N =* 21)	4 (19.05)	9 (42.86)	4 (19.05)	4 (19.05)	17 (80.95)
Group C (*N =* 19)	4 (21.05)	2 (10.53)	3 (15.79)	10 (52.63)	9 (47.37)
Group D (*N =* 20)	1 (5.00)	8 (40.00)	6 (30.00)	5 (25.00)	15 (75.00)

Overall efficiency = (no. of patients recovered +no. of patients evident+ no. of patients effective)/no. of the group.

### Comparison of results of secondary indicators before treatment between groups

As shown in Tables 3–6, there were no statistical differences in the VAS scores, CSTI scores, ODI scores, and NPQ scores between the groups before treatment (*P* > 0.05).

**Table 3:** Comparison of VAS scores between groups before treatment.

**Statistics**	**Group A**	**Group B**	**Group C**	**Group D**
*n*	34	21	19	20
Mean (SD)	5.09 (1.56)	4.81 (2.09)	4.84 (1.80)	4.90 (2.00)
Median (P_25_, P_75_)	5.00 (4.00, 6.00)	5.00 (3.00, 6.00)	5.00 (3.00, 7.00)	5.00 (3.00, 6.50)
Min, Max	2.00, 8.00	2.00, 10.00	2.00, 7.00	2.00, 9.00

**Table 4:** Comparison of chronic tissue injury scores between groups before treatment.

**Statistics**	**Group A**	**Group B**	**Group C**	**Group D**
*n*	34	21	19	20
Mean (SD)	2.15 (1.48)	2.05 (1.36)	1.63 (1.12)	1.95 (1.15)
Median (P_25_,P_75_)	2.00 (1.00, 3.00)	2.00 (1.00, 3.00)	1.00 (1.00, 2.00)	2.00 (1.50, 2.50)
Min,Max	0.00, 7.00	0.00, 5.00	0.00, 4.00	0.00, 4.00

**Table 5:** Comparison of ODI scores between groups before treatment.

**Statistics**	**Group A**	**Group B (*N =* 21)**	**Group C**	**Group D**
*n*	21	14	12	17
Mean (SD)	7.76 (7.18)	8.86 (5.08)	8.0 (3.64)	7.18 (5.17)
Median (P_25_,P_75_)	7.00 (3.00, 10.00)	7.50 (5.00, 13.00)	9.00 (5.00, 10.00)	6.00 (3.00, 8.00)
Min, Max	1.00, 34.00	3.00, 19.00	2, 14.00	1.00, 21.00

**Table 6:** Comparison of NPQ scores between groups before treatment.

**Statistics**	**Group A**	**Group B**	**Group C**	**Group D**
*n*	15	7	8	5
Mean (SD)	28.19 (12.23)	24.85 (9.72)	22.40 (9.99)	33.26 (17.64)
Median (P25,P75)	25.00 (18.75, 41.67)	22.22 (16.67, 36.11)	23.61 (15.28, 29.51)	30.56 (19.44, 34.38)
Min,Max	12.50, 50.00	11.11, 37.50	6.25, 36.11	19.44, 62.50

One patient with neck, shoulder, and back pain was included in group C, and two patients with neck, shoulder, and low back pain were included in groups A and D. Therefore, the ODI and NPQ scores were present and included in the statistics.

### Intragroup comparison results of secondary indicators before and after treatment in each group

As shown in Supplementary Figures 2–5, the results showed that all four evaluation indicators in group A and group B were significantly improved, only VAS scores in group C were significantly improved, and three evaluation indicators in group D were significantly improved.

### Intergroup comparison of changes in secondary indicators after treatment in each group from baseline

The results show that, as shown in Table 7, after 2 weeks of treatment, all secondary indicators in each group decreased to a certain extent compared with the baseline, and there was no statistical difference between groups A, B, and D (*P* > 0.05). All groups have an obvious curative effect. As shown in Supplementary Figures 6, 7, there were significant differences in VAS scores and CSTI scores before and after treatment in group A vs. group C (*P* = 0.0145, *P* = 0.0005); there were significant differences in CSTI scores in group B vs. group C before and after treatment (*P* = 0.0280).

**Table 7:** Intergroup comparison of changes in secondary indicators from baseline for each group.

**Indicators**	**Statistics**	**Group A**	**Group B**	**Group C**	**Group D**
VAS scores					
	*n*	34	21	19	20
	Mean (SD)	−2.71 (1.85)	−2.38 (1.47)	−1.53 (1.78)	−2.05 (1.32)
CSTI scores					
	*n*	34	21	19	20
	Mean (SD)	−1.53 (1.52)	−1.14 (1.06)	−0.32 (0.58)	−1.00 (0.97)
ODI scores					
	*n*	21	14	12	17
	Mean (SD)	−4.19 (5.28)	−4.79 (4.63)	−1.50 (2.24)	−1.56 (3.67)
NPQ scores					
	*n*	15	7	8	5
	Mean (SD)	−17.06 (11.11)	−15.87 (11.78)	−10.94 (10.89)	−19.51 (14.38)

One patient with neck, shoulder, and back pain was included in group C, and two patients with neck, shoulder, and back pain were included in groups A and D. Therefore, the ODI and NPQ scores were present and included in the statistics.

### Intragroup comparison of secondary indicators before and after treatment in patients with different pain sites in groups A and B

As shown in Table 8, patients with back pain had significant improvements in all indicators in both groups. However, for patients with neck and shoulder pain, group A improved more evaluation indicators, as shown in Table 9.

**Table 8:** Intragroup comparison of secondary indicators before and after treatment in patients with back pain.

**Treatment groups**	**Indicators**	**Statistics**	**Pre-treatment**	**Post-treatment**	***P-value***
Group A (*N =* 21)					
	VAS scores	*n*	21	21	
		Mean (SD)	4.76 (1.76)	2.14 (1.15)	<0.0001
	CSTI scores	*n*	21	21	
		Mean (SD)	2.10 (1.76)	0.52 (0.60)	0.0004
	ODI scores	*n*	21	21	
		Mean (SD)	7.76 (7.18)	3.57 (2.87)	0.0174
Group B (*N =* 14)					
	VAS scores	*n*	14	14	
		Mean (SD)	5.07 (2.09)	2.64 (1.60)	0.0019
	CSTI scores	*n*	14	14	
		Mean (SD)	2.07 (1.54)	0.86 (0.77)	0.0140
	ODI scores	*n*	14	14	
		Mean (SD)	8.86 (5.08)	4.07 (3.50)	0.0074

**Table 9:** Intragroup comparison of secondary indicators before and after treatment in patients with neck and shoulder pain.

**Treatment groups**	**Indicators**	**Statistics**	**Pre-treatment**	**Post-treatment**	***P-value***
Group A (*N =* 15)					
	VAS scores	*n*	15	15	
		Mean (SD)	5.47 (1.19)	2.73 (1.67)	<0.0001
	CSTI scores	*n*	15	15	
		Mean (SD)	2.33 (0.90)	0.73 (0.80)	<0.0001
	NPQ scores	*n*	15	15	
		Mean (SD)	28.19 (12.23)	11.13 (9.77)	0.0002
Group B (*N =* 7)					
	VAS scores	*n*	7	7	
		Mean (SD)	4.29 (2.14)	2.00 (2.08)	0.0655
	CSTI scores	*n*	7	7	
		Mean (SD)	2.00 (1.00)	1.00 (1.00)	0.0859
	NPQ scores	*n*	7	7	
		Mean (SD)	24.85 (9.72)	8.98 (8.14)	0.0062

## Discussion

There are various treatment options for MPS, but there is still no optimal one (2). To summarize a simple, convenient, and popularized regimen, this study explored the safety and efficacy of different non-invasive treatment regimens for MPS. We found that Loxoprofen Sodium Cataplasm combined with ESWT and TENS therapy can help relieve pain in patients with MPS, relieve CSTI, and facilitate the recovery of patients' daily life functions. At the same time, this program has certain advantages over other control groups. NSAIDs topical patch is an alternative to oral medications, which has a local effect on the painful area and reduces the systemic impact (23). Even if adverse reactions such as allergic reactions occur, the allergen can be removed immediately by removing the cataplasm. ESWT and TENS are widely used in clinical practice because of their non-invasiveness, convenient operation, and short treatment time. The treatment of Loxoprofen Sodium Cataplasm combined with ESWT and TENS can be used safely even in areas with underdeveloped sanitary conditions, reducing the number of patients visiting the hospital, improving patient compliance with treatment, and facilitating widespread promotion.

We found that after 2 weeks of treatment, the different non-invasive treatment regimens were effective in terms of overall efficiency and evaluation of secondary indicators. There was no difference in the overall efficiency between group A, group B, and group D. Group C, the physiotherapy group, has a lower overall efficiency, which suggested that physiotherapy was not suitable for the clinical treatment of MPS alone. In addition, we found that there was no significant difference in the overall efficiency between group A and group B, indicating that after the external application of Loxoprofen Sodium Cataplasm, even combined ESWT and TENS would not significantly improve the overall efficiency of the treatment. This may suggest that the pain of MPS can be effectively relieved after the Loxoprofen Sodium Cataplasm is applied alone. Whether the Flurbiprofen Cataplasm alone can achieve the same effect remains to be further studied.

When analyzing secondary indicators for intragroup comparison, we found consistent and significant improvements in VAS scores, CSTI scores, ODI scores, and NPQ scores after loxoprofen, with or without physical therapy. With physiotherapy alone, only one secondary indicator, VAS scores, was improved, which did not relieve soft tissue injury and limitations in daily life. In addition, the Flurbiprofen Cataplasm combined with the physiotherapy group showed effectiveness in pain, CSTI, and neck and shoulder pain, but the improvement of ODI scores was not significant, suggesting that this regimen is not suitable for patients with back pain. In addition, when comparing between groups, we found that Loxoprofen Sodium Cataplasm combined with physiotherapy significantly improved CSTI and VAS scores compared with physical therapy alone, and Loxoprofen Sodium Cataplasm alone was associated with significant improvements in CSTI scores, which shows that Loxoprofen Sodium Cataplasm has a significant therapeutic effect on MPS, and a single application can significantly improve the quality of daily life of patients, which is helpful for patients' work and life.

To further analyze the efficacy of Loxoprofen Sodium Cataplasm alone and Loxoprofen Sodium Cataplasm combined with physiotherapy on MPS patients with different pain sites, we conducted intragroup comparisons of secondary evaluation indicators for patients with different pain sites in the two regimens. We found that compared with before treatment, Loxoprofen Sodium Cataplasm improved the secondary evaluation indicators in patients with back pain with or without combined physiotherapy. The possible reason is that the efficacy of ESWT and TENS physical therapy is dose-dependent (24, 25), and higher treatment intensity corresponds to more obvious efficacy. In patients with back pain, due to the deep location of myofascial trigger points (26), when we use the same ESWT and TENS treatment parameters as those of the neck and shoulders to treat back pain, the degree of tissue stimulation is relatively weak, so we get this result. However, in patients with neck and shoulder pain, Loxoprofen Sodium Cataplasm combined with physiotherapy had more significant improvements in secondary evaluation indicators.

This study has several limitations. First of all, although outpatients have been guided by doctors on daily-related precautions and exercise methods after patients leave the outpatient clinic, their exercise and rehabilitation cannot be objectively evaluated. We did not perform a systematic neurological examination of the patients, such as EMG or MRC scales. Especially in patients with MPS in the neck and shoulders, when the pain occurs, the activities of the neck and shoulders are limited. After ESWT and TENS treatment, the immediate improvement effect is obvious (27). The increase in joint activity also has a positive effect on relieving musculoskeletal pain (28, 29), which has a certain impact on the evaluation of the results of the study. Second, this study did not conduct relevant research on the long-term efficacy of patients. Finally, the sample size is small. It will be necessary to increase the sample size and long-term follow-up in the future.

## Conclusion

Loxoprofen Sodium Cataplasm combined with physiotherapy can help relieve pain in patients with MPS, improve CSTI, and facilitate the recovery of patients' daily life functions. The treatment regimen in the control group has certain advantages.

## Data availability statement

The original contributions presented in the study are included in the article/Supplementary material, further inquiries can be directed to the corresponding author/s.

## Ethics statement

The studies involving human participants were reviewed and approved by the Clinical Trial Ethics Committee of the Third Xiangya Hospital of Central South University (No. 21136). The patients/participants provided their written informed consent to participate in this study. Written informed consent was obtained from the individual(s) for the publication of any potentially identifiable images or data included in this article.

## Author contributions

ZW conceived and designed the experiments. XZ and XL wrote the paper. ZW and XL interpreted and analyzed the data. ZW, XZ, and XL performed the experiments and acquired the data. XL performed the statistical analysis. ZW and DH had full access to all the data in the study and had final responsibility for the decision to submit it for publication. All authors stated were involved in the critical revision of the manuscript and approved the final version of the article, including the authorship list.

## Funding

This study was supported by Hunan Jiudian Pharmaceutical Co., Ltd. which made the Loxoprofen Sodium Cataplasm available to the investigators. Funders of the study had no role in study design, data analysis, data interpretation, or writing of the report.

## Conflict of interest

The authors declare that the research was conducted in the absence of any commercial or financial relationships that could be construed as a potential conflict of interest.

## Publisher's note

All claims expressed in this article are solely those of the authors and do not necessarily represent those of their affiliated organizations, or those of the publisher, the editors and the reviewers. Any product that may be evaluated in this article, or claim that may be made by its manufacturer, is not guaranteed or endorsed by the publisher.
