# Supplementary material for: Preliminary evidence of safety and effectiveness of Loxoprofen Sodium Cataplasm combined with physiotherapy for myofascial pain syndrome treatment: A randomized controlled pilot clinical trial
Source: Front Neurol. 2022 Nov 22;13:998327. doi: 10.3389/fneur.2022.998327 (PMC9724624; doi:10.3389/fneur.2022.998327)
Supplement: Supplementary file 1 [file Data_Sheet_1.docx]

Supplementary Material

# Supplementary Figures


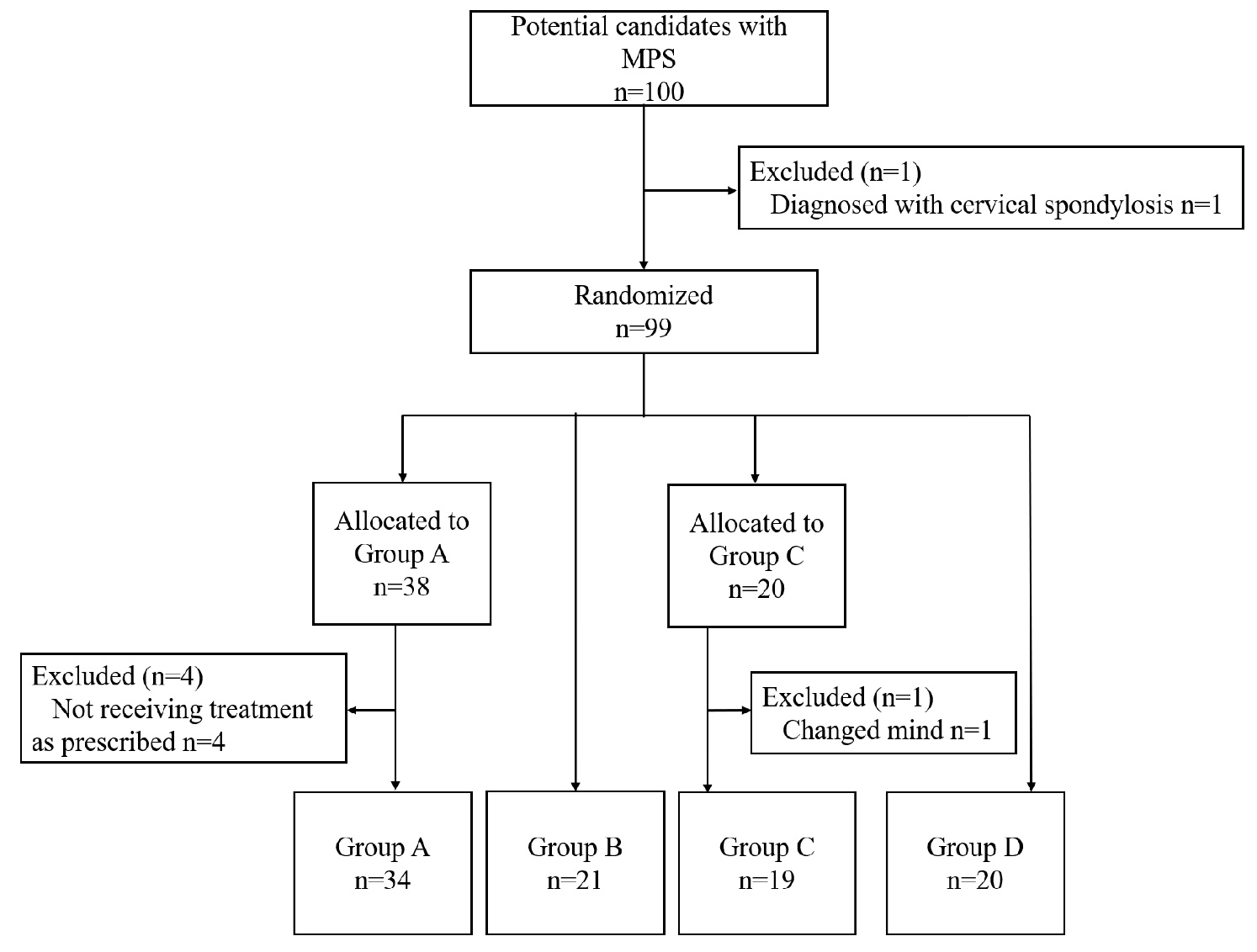


Figure 1. Flow chart of patient selection.

# Supplementary

**
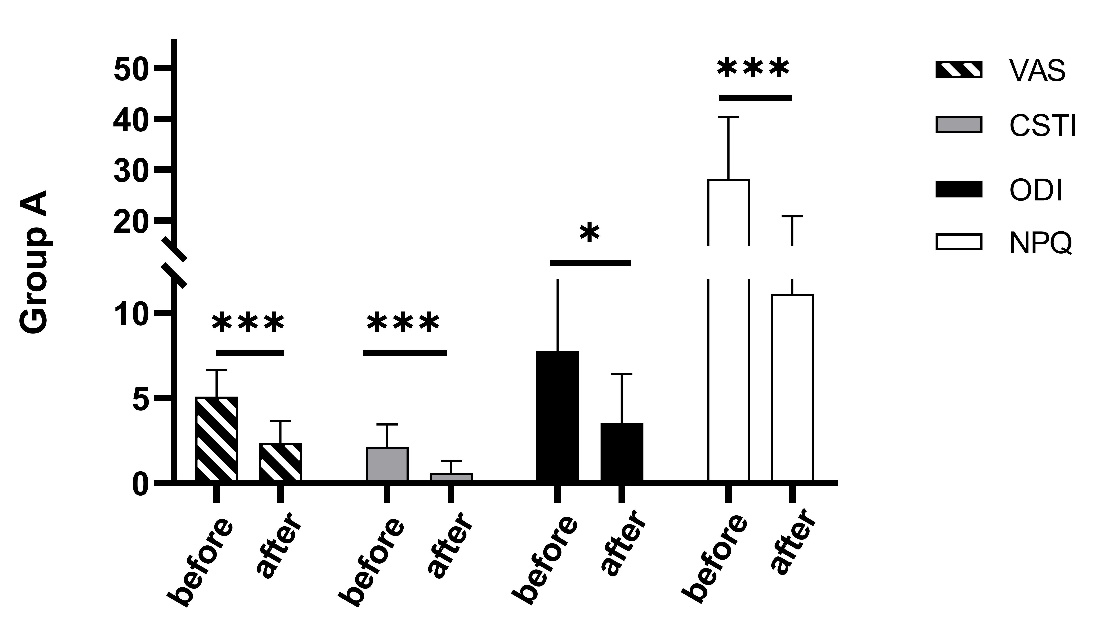
**

**Supplementary Figure 2.** Differences before and after treatment in Group A


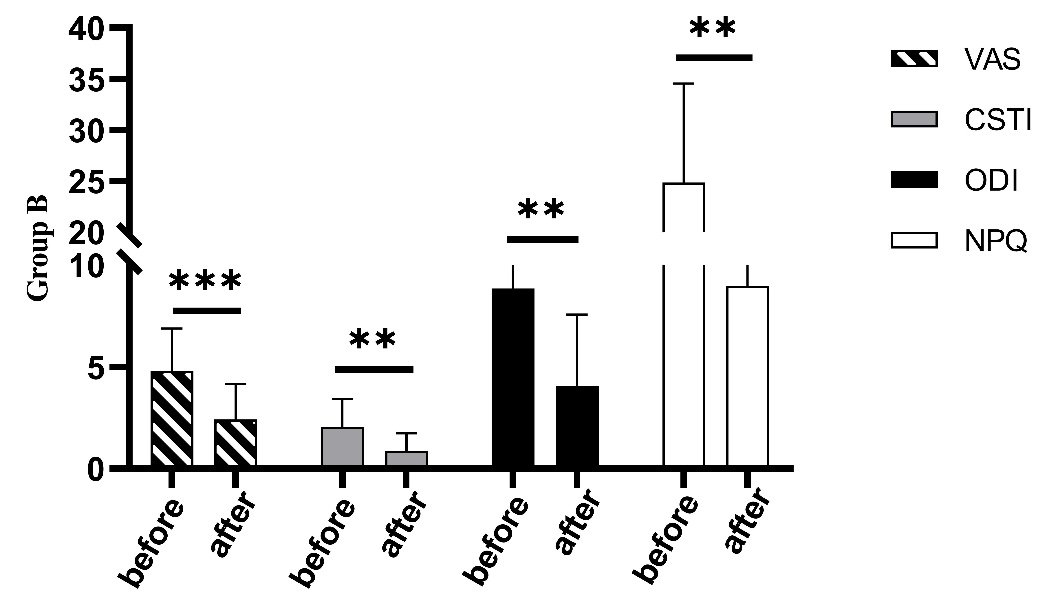


**Supplementary Figure 3.** Differences before and after treatment in Group B


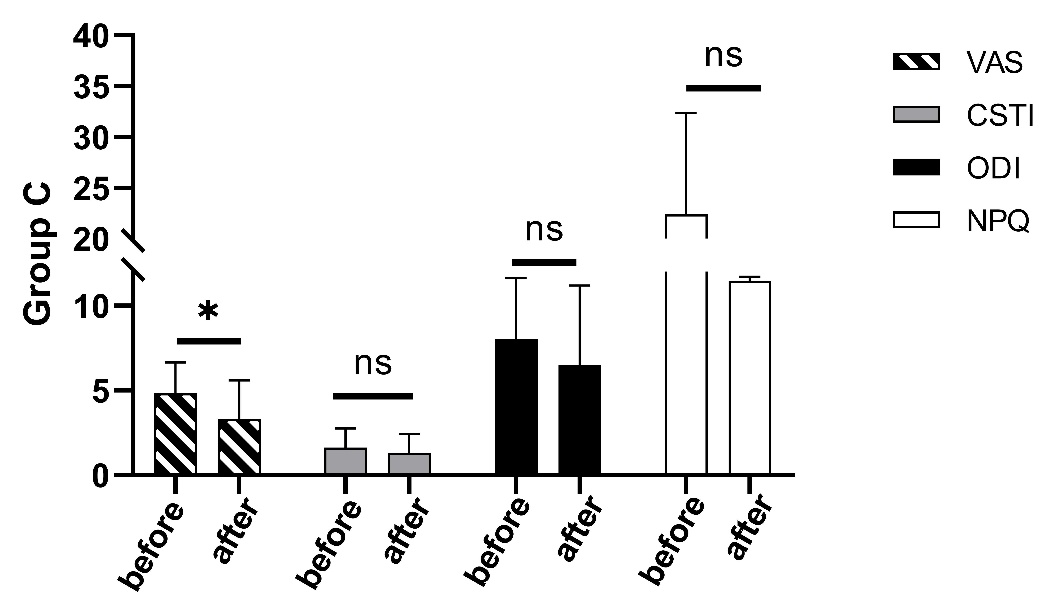


**Supplementary Figure 4.** Differences before and after treatment in Group C


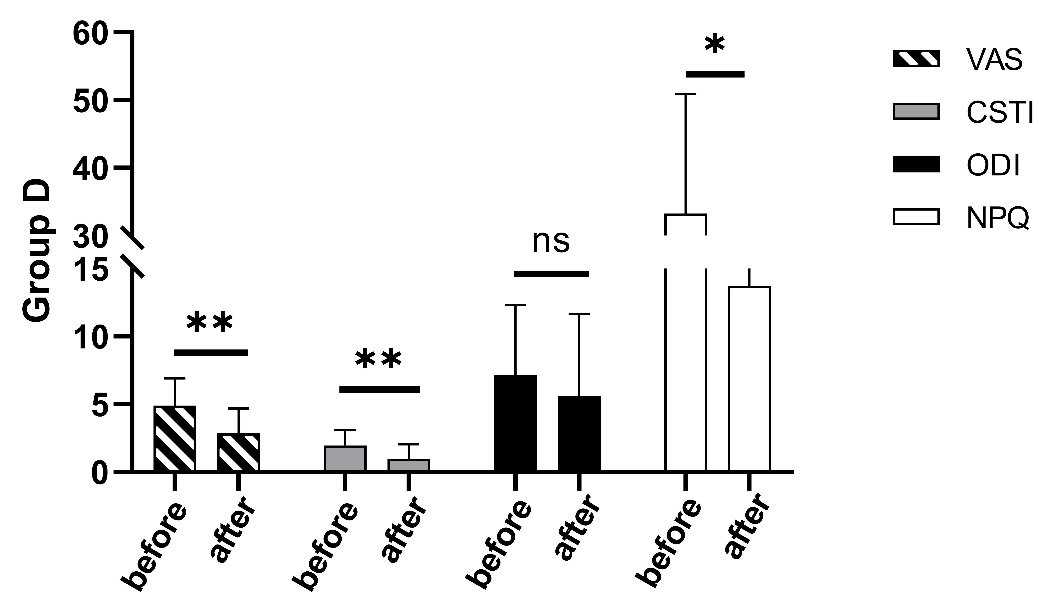


**Supplementary Figure 5.** Differences before and after treatment in Group D


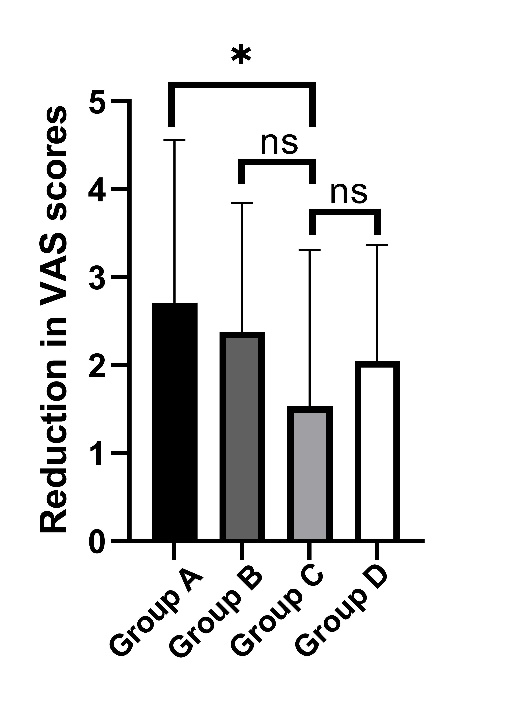


**Supplementary Figure 6.** Reduction in VAS scores


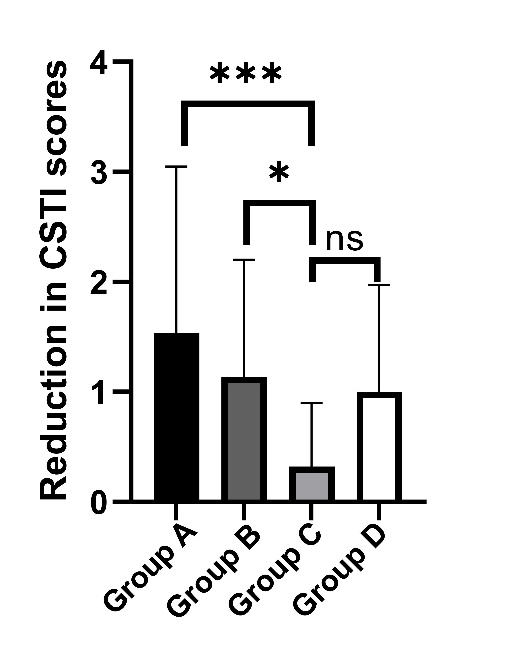


**Supplementary Figure 7.** Reduction in CSTI scores

Table S1 Effective number of young adult patients in each group (overall efficiency) n (%)

| Test groups | recover | evident | effective | effectless | overall efficiency |
| --- | --- | --- | --- | --- | --- |
| Group A（n=12） | 3（25.00） | 5（41.67） | 4（33.33） | 0（0.00） | 12（100.00） |
| Group B（n=12） | 4（33.33） | 4（33.33） | 3（35.00） | 1（8.33） | 11（91.67） |
| Group C（n=9） | 1（11.11） | 2（22.22） | 2（22.22） | 4（44.44） | 5（55.56） |
| Group D（n=9） | 0（0.00） | 4（44.44） | 5（55.56） | 0（0.00） | 9（100.00） |

Table S2 Intergroup comparison of changes in secondary indicators from baseline for each group (young adult patients)

| Indicators | Statistics | Group A | Group B | Group C | Group D |
| --- | --- | --- | --- | --- | --- |
|  |  |  |  |  |  |
| VAS scores |  |  |  |  |  |
|  | n（nmiss） | 12(0) | 12(0) | 9(0) | 9(0) |
|  | Mean（SD） | -2.83（2.04） | -2.42（1.08） | -1.44（1.33） | -2.11（0.78） |
| CSTI scores |  |  |  |  |  |
|  | n（nmiss） | 12(0) | 12(0) | 9(0) | 9(0) |
|  | Mean（SD） | -1.33（1.15） | -1.33（1.07） | -0.33（0.50） | -1.22（1.09） |
| ODI scores |  |  |  |  |  |
|  | n（nmiss） | 11（1） | 9（3） | 7（2） | 9（0） |
|  | Mean（SD） | -3.91（3.24） | -5.11（4.73） | -1.57（2.70） | -0.44（3.00） |
| NPQ scores |  |  |  |  |  |
|  | n（nmiss） | 2（10） | 3（9） | 2（7） | 1（8） |
|  | Mean（SD） | -21.53（12.77） | -20.37（14.85） | -15.28（5.89） | -8.33（—） |

Table S3 Intra-group comparison of secondary indicators before and after treatment in young adult patients

| Treatment groups | Indicators | Statistics | Pre-treatment | Post-treatment | *P-value* |
| --- | --- | --- | --- | --- | --- |
| Group A（N=12） |  |  |  |  |  |
|  | VAS scores | n（nmiss） | 12（0） | 12（0） | <0.0001 |
|  |  | Mean（SD） | 4.58（1.73） | 1.75（1.06） |  |
|  | CSTI scores | n（nmiss） | 12（0） | 12（0） | 0.0015 |
|  |  | Mean（SD） | 1.92（1.16） | 0.58（0.51） |  |
|  | ODI scores | n（nmiss） | 11（1） | 11（1） | 0.0112 |
|  |  | Mean（SD） | 7.55（4.06） | 3.64（2.25） |  |
|  | NPQ scores | n（nmiss） | 2（10） | 2（10） | 0.1399 |
| Group B（N=12） |  | Mean（SD） | 21.53（12.77） | 0.00（0.00） |  |
|  | VAS scores | n（nmiss） | 12（0） | 12（0） | 0.0016 |
|  |  | Mean（SD） | 4.17（1.70） | 1.75（1.60） |  |
|  | CSTI scores | n（nmiss） | 12（0） | 12（0） | 0.0120 |
|  |  | Mean（SD） | 2.17（1.47） | 0.83（0.83） |  |
|  | ODI scores | n（nmiss） | 9（3） | 9（3） | 0.0145 |
|  |  | Mean（SD） | 7.56（5.15） | 2.44（2.19） |  |
|  | NPQ scores | n（nmiss） | 3（9） | 3（9） | 0.0545 |
| Group C（N=12） |  | Mean（SD） | 27.43（10.43） | 7.06（7.92） |  |
|  | VAS scores | n（nmiss） | 9（0） | 9（0） | 0.1365 |
|  |  | Mean（SD） | 4.89（1.69） | 3.44（2.19） |  |
|  | CSTI scores | n（nmiss） | 9（0） | 9（0） | 0.6113 |
|  |  | Mean（SD） | 1.78（1.39） | 1.44（1.33） |  |
|  | ODI scores | n（nmiss） | 7（2） | 7（2） | 0.3390 |
|  |  | Mean（SD） | 7.43（2.37） | 5.86（3.44） |  |
|  | NPQ scores | n（nmiss） | 2（7） | 2（7） | 0.1778 |
| Group D（N=12） |  | Mean（SD） | 22.22（3.93） | 6.94（9.82） |  |
|  | VAS scores | n（nmiss） | 9（0） | 9（0） | 0.0350 |
|  |  | Mean（SD） | 4.56（2.19） | 2.44（1.67） |  |
|  | CSTI scores | n（nmiss） | 9（0） | 9（0） | 0.0249 |
|  |  | Mean（SD） | 2.00（1.22） | 0.78（0.83） |  |
|  | ODI scores | n（nmiss） | 9（0） | 9（0） | 0.8409 |
|  |  | Mean（SD） | 4.78（3.42） | 4.33（5.57） |  |
|  | NPQ scores | n（nmiss） | 1（8） | 1（8） | <0.0001 |
|  |  | Mean（SD） | 19.44（—） | 11.11（—） |  |
